# Supplementary material for: The spatial RNA integrity number assay for in situ evaluation of transcriptome quality
Source: Commun Biol. 2021 Jan 8;4:57. doi: 10.1038/s42003-020-01573-1 (PMC7794352; doi:10.1038/s42003-020-01573-1)
Supplement: Supplementary file 2 — Supplementary Information [file 42003_2020_1573_MOESM2_ESM.pdf]

Supplementary Information

The spatial RNA integrity number (sRIN) assay for *in situ* evaluation of transcriptome quality

Linda Kvastad[1], Konstantin Carlberg[1*], Ludvig Larsson[1*], Eva Gracia Villacampa[1*], Alexander Stuckey[1], Linnea Stenbeck[1], Annelie Mollbrink[1], Margherita Zamboni[2], Jens Peter Magnusson[2,3], Elisa Basmaci[4], Alia Shamikh[4], Gabriella Prochazka[4], Anna-Lena Schaupp[5], Åke Borg[6], Lars Fugger[5], Monica Nistér[4] and Joakim Lundeberg[1†].

[1]Science for Life Laboratory, KTH - Royal Institute of Technology (KTH), SE-171 65, Solna, Sweden.
[2]Department of Cell and Molecular Biology, Karolinska Institutet, Sweden.
[3]Current affiliation: Bioengineering Department, Stanford University, Stanford, USA
[4]Department of Oncology-Pathology, Karolinska Institutet, Stockholm, Sweden; Clinical Pathology and Cytology, Karolinska University Hospital, Stockholm, Sweden.
[5]Oxford Centre for Neuroinflammation, Nuffield Department of Clinical Neurosciences, MRC Human Immunology Unit, Weatherall Institute of Molecular Medicine, John Radcliffe Hospital, University of Oxford, Oxford, UK.
[6]Division of Oncology and Pathology, Department of Clinical Sciences Lund, Lund University, Sweden.

[†]Corresponding author

* Authors contributed equally

Corresponding author:
Joakim Lundeberg
Science for Life Laboratory
KTH Royal Institute of Technology
Department of Gene Technology
SE-171 65 Solna, Sweden
joakim.lundeberg@scilifelab.se

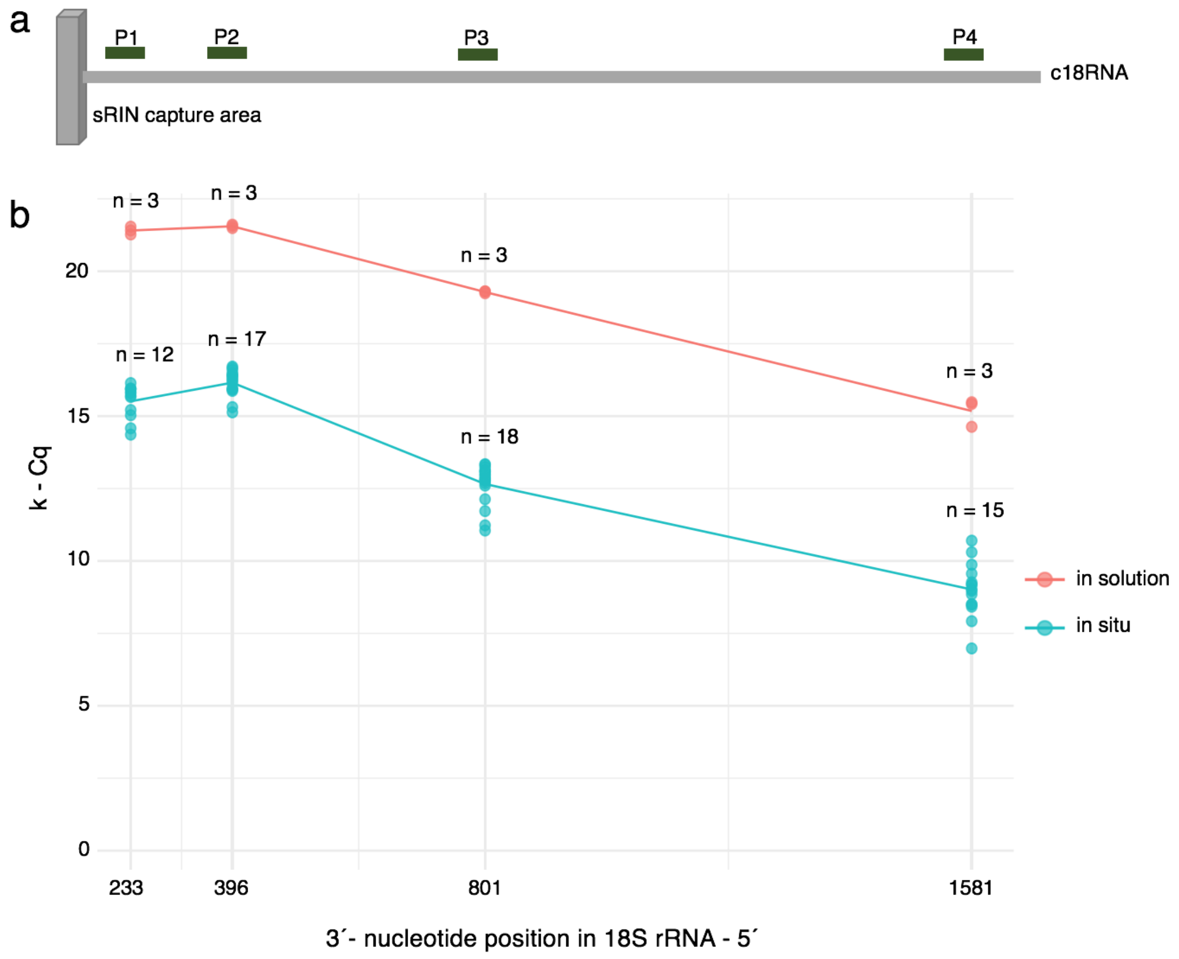

Supplementary Figure 1. Gene body coverage of 18S rRNA used for generation of c18RNA *in situ* on a fully coated capture area. We estimated the gene body coverage based on the mean quantification cycle (Cq) value, where k = 30 was set as a cut-off Cq value for positive signals compared to negative controls. **a** By using primers where one primer sequence in each pair corresponded to one of the four Probes (P1-P4) used in the sRIN assay for sequential hybridization (Supplementary Table 1), each hybridizing to different sites of the c18RNA corresponding to different positions into the 18S rRNA transcript. **b** To determine the gene body coverage of the c18RNA used in the sRIN assay, we compared c18RNA synthesized *in situ* from mouse olfactory bulb and cleaved off from the assay's capture area with c18RNA synthesized in solution from total RNA (LNCaP) and performed qPCR (the number of technical replicates are shown in Supplementary Figure 1b). We observed a full-length gene body coverage of the 18S rRNA transcript with a slight bias towards the 3' end. By comparing Cq values from c18RNA generated *in situ* and in solution, we observed similar gene body coverage patterns for all four probe positions.

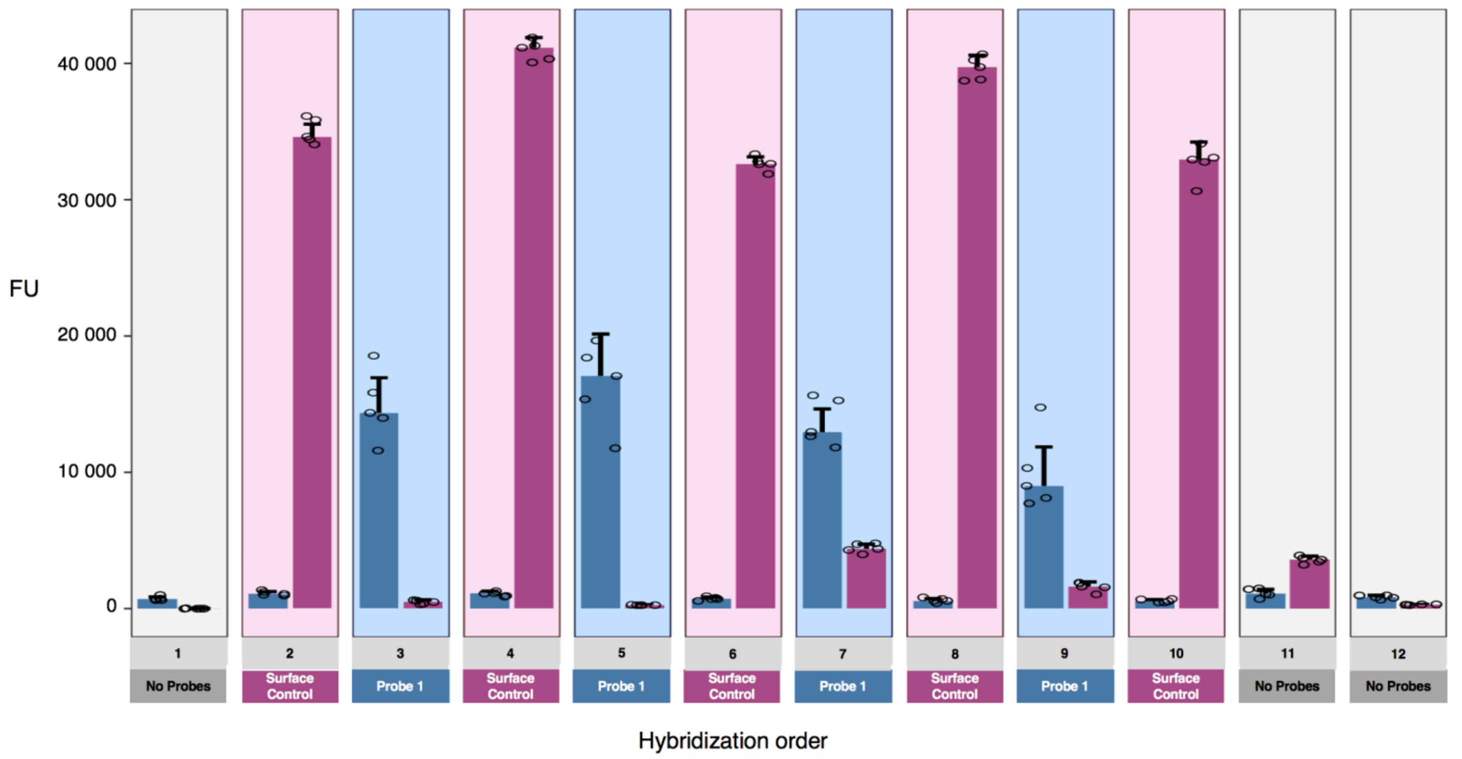

Supplementary Figure 2. Robustness and durability of c18RNA footprint. The sRIN assay applied to a mouse olfactory bulb. Sequential probe hybridization, chronologically ordered from left to right, using Cy3 (blue) labelled Probe 1 (P1), Cy5 (purple) labelled surface probe as control or no probes, where P1 signal indicates presence of synthesized c18RNA and surface control probe signal indicates presence of pre-printed DNA oligonucleotides for 18S rRNA capture. We recorded signals from both the Cy3 and Cy5 channel for each round, measuring the fluorescence units (FU) signal from the same locations (n=5, biological replicates) each time, bars represent mean FU and error bars represent standard deviation. We selected P1 for this experiment because it is also used to normalize the data from all Probes (P1-P4) used for hybridization when generating sRIN heat maps. The surface probe served as a quality check of the DNA oligonucleotide printed glass slide area. This enabled us to visualize all areas of possible 18S rRNA capture, which we used as an internal control to test for areas of possible false negative signals in the final imaging step of the sRIN assay. Some residual surface probe signal could sometimes be viewed after the slide with c18RNA footprint was kept dried overnight. To address this we incorporated a short rehydration step in the sRIN assay protocol when choosing to perform the sequential hybridizations over separate days.

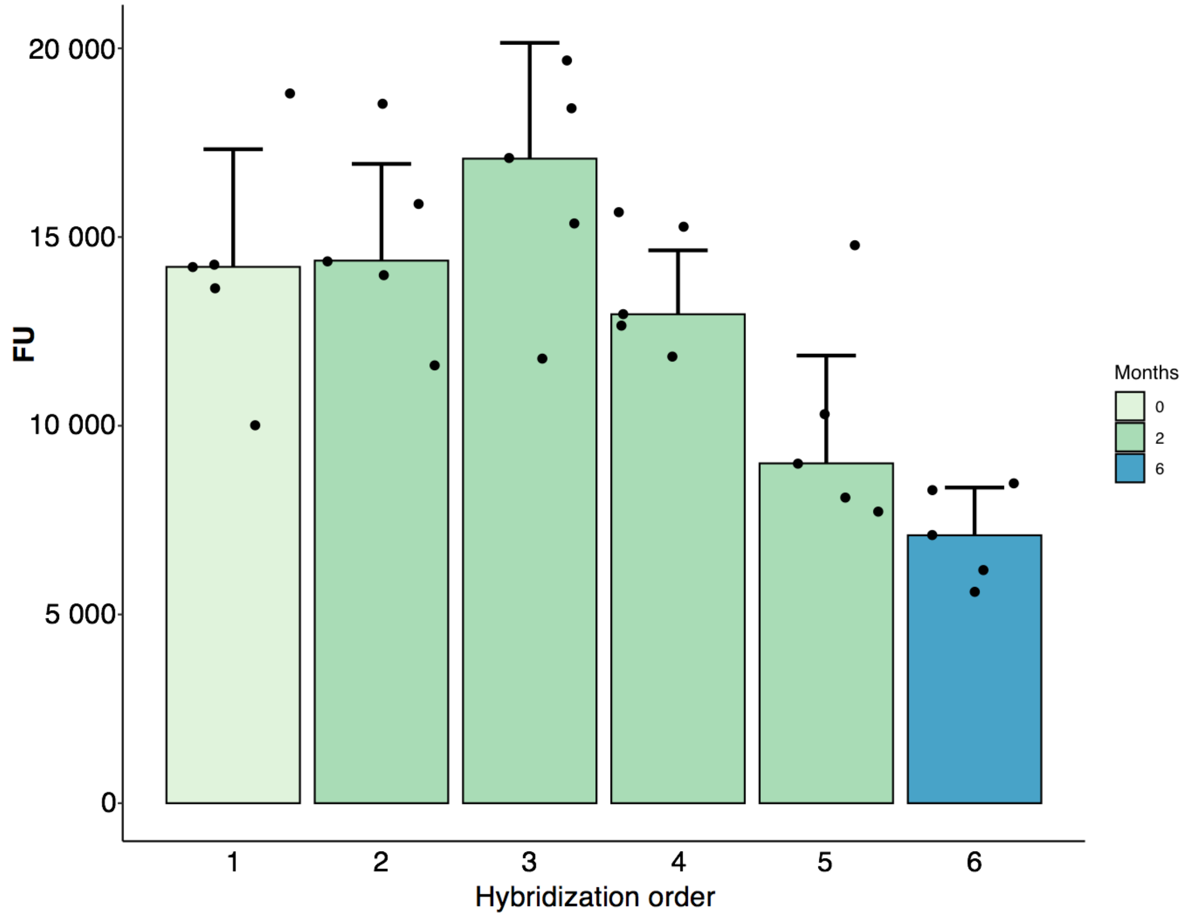

Supplementary Figure 3. Stability of c18RNA footprint over time. Shown is the FU signal generated from a c18RNA footprint from the same areas (n=5, biological replicates) using the same sRIN slide after re-hybridization with P1, bars represent standard deviation. Hybridizations were done over 6 months, most were performed close together after 2 months. The sRIN slide was stored dried at low humidity in a desiccation chamber at room temperature. Bars represent mean FU (FU = Fluorescence Units) and error bars represent standard deviation

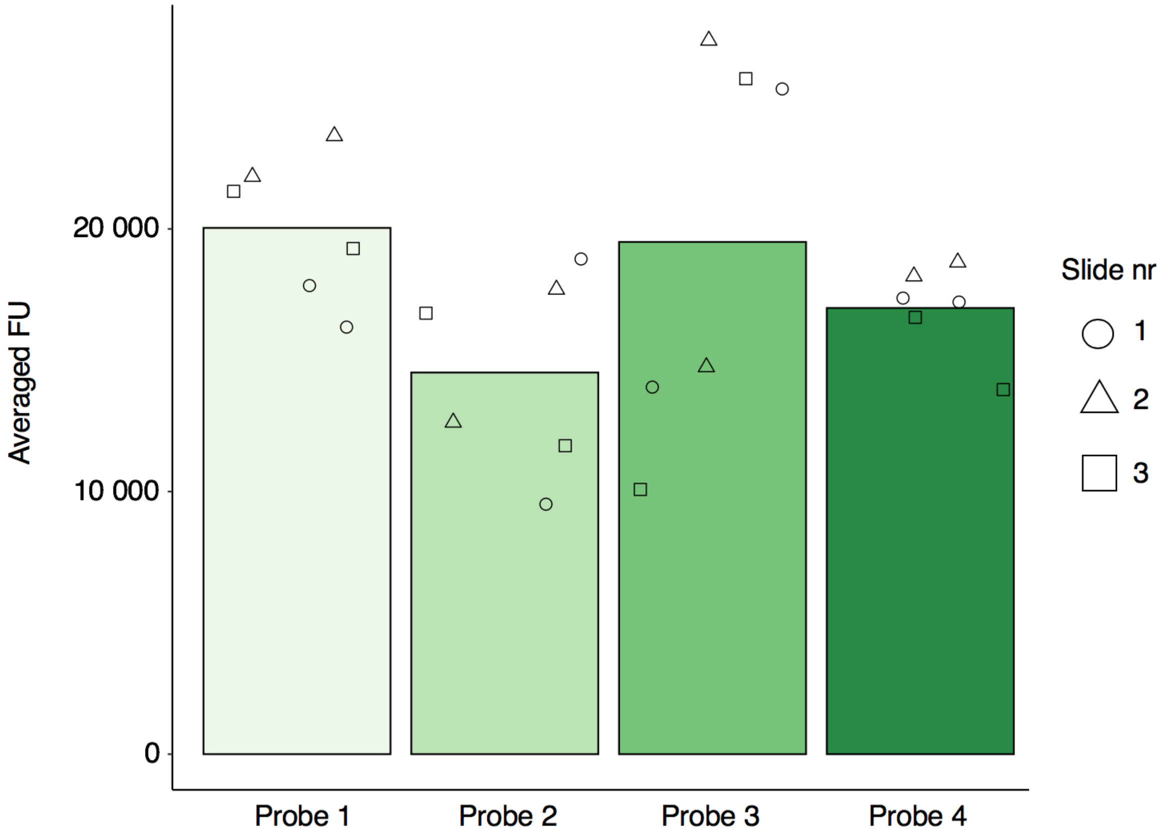

Supplementary Figure 4. Similar hybridization efficiency of probes.
All probes in the sRIN assay, used to estimate the sRIN values, were tested for their hybridization efficiency towards their complementary strand on a capture area. Bars represent averaged FU (FU = Fluorescence Units) from different capture areas (n = 6, technical replicates) and from different control probe slides (n = 3).

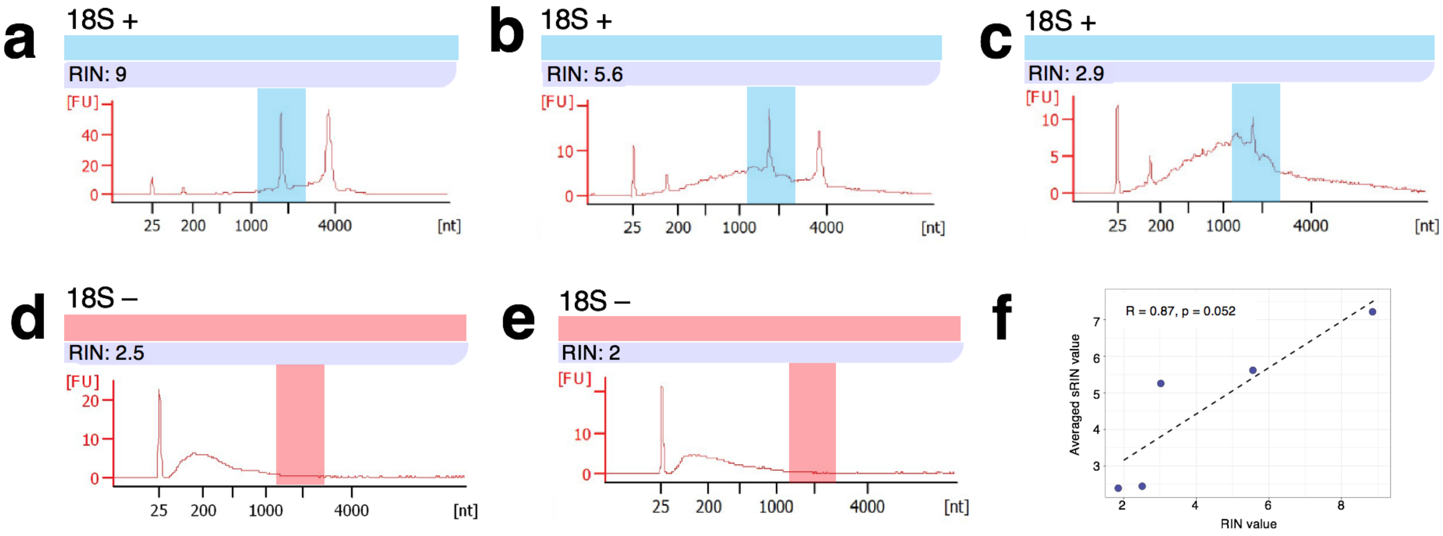

Supplementary Figure 5. RNA integrity measured in bulk and comparing sRIN with RIN. Total RNA fragmented to different RIN values, measured on the Bioanalyzer system. The sample with a RIN value of 5.6 is a 1:4 (v/v) mixture of the samples with RIN values of 9 and 2.9 respectively. We divided the samples into two groups, based on (**a - c**) the presence (18S+, blue) or (**d - e**) absence (18S—, red) of the 18S rRNA peak. **f** Pearson correlation coefficient and p-value between averaged sRIN and RIN (5=n biological replicates) were calculated to r = 0.87 and p = 0.052.

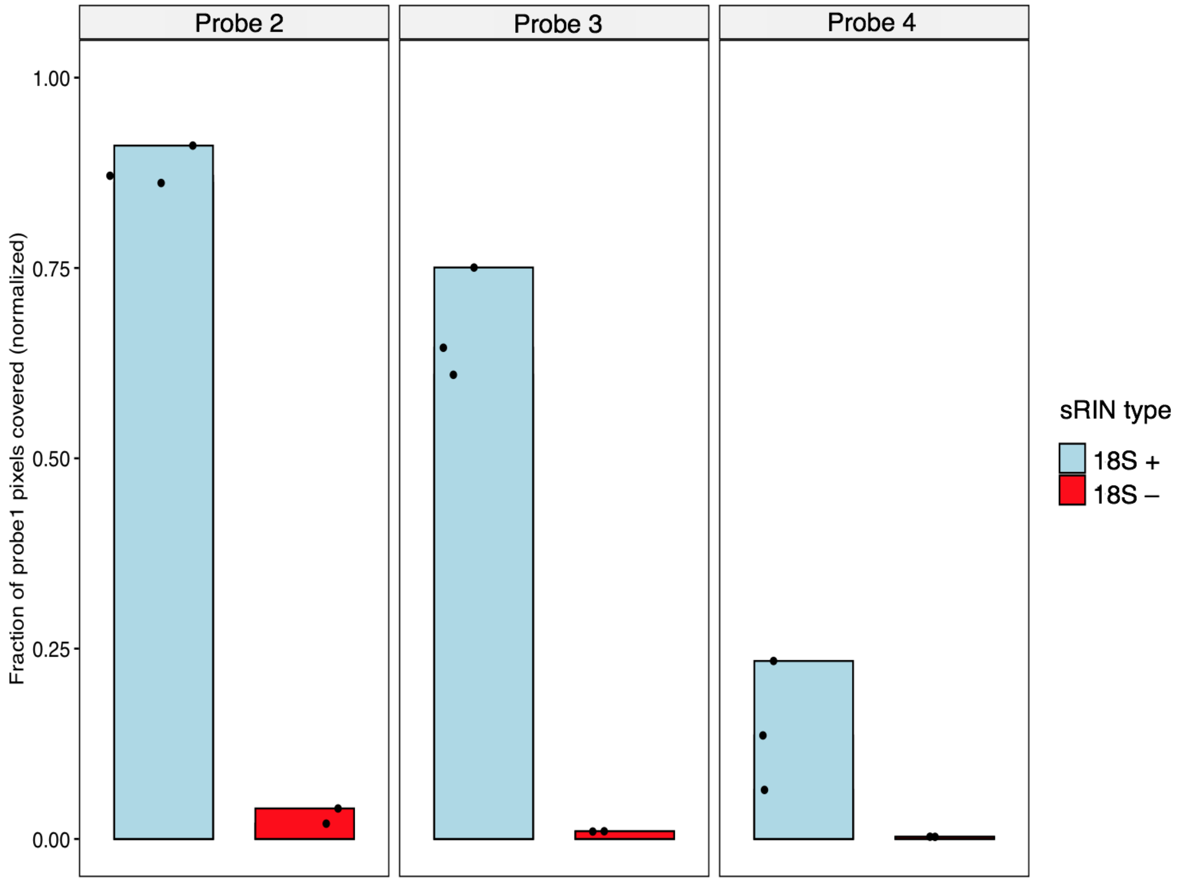

Supplementary Figure 6. Detection with sRIN assay shows strong positive signal only from 18S rRNA positive samples. We divided total RNA samples into two groups based on the presence (18S+) or absence (18S-) of a visible 18S rRNA peak in their electropherograms. We then compared the fluorescence signals observed with the sRIN assay of samples from these two groups. A clear difference was observed in signals for P2-P4 between samples who were 18S+ (n = 3, biological replicates) or 18S- (n = 2, biological replicates) when using the sRIN assay, consistent with the sRIN assay's 18S rRNA specificity. The P1 probe was used for normalization of P2-P4. Bars represent the mean fraction of normalized Probe 1 pixels covered on the capture area.

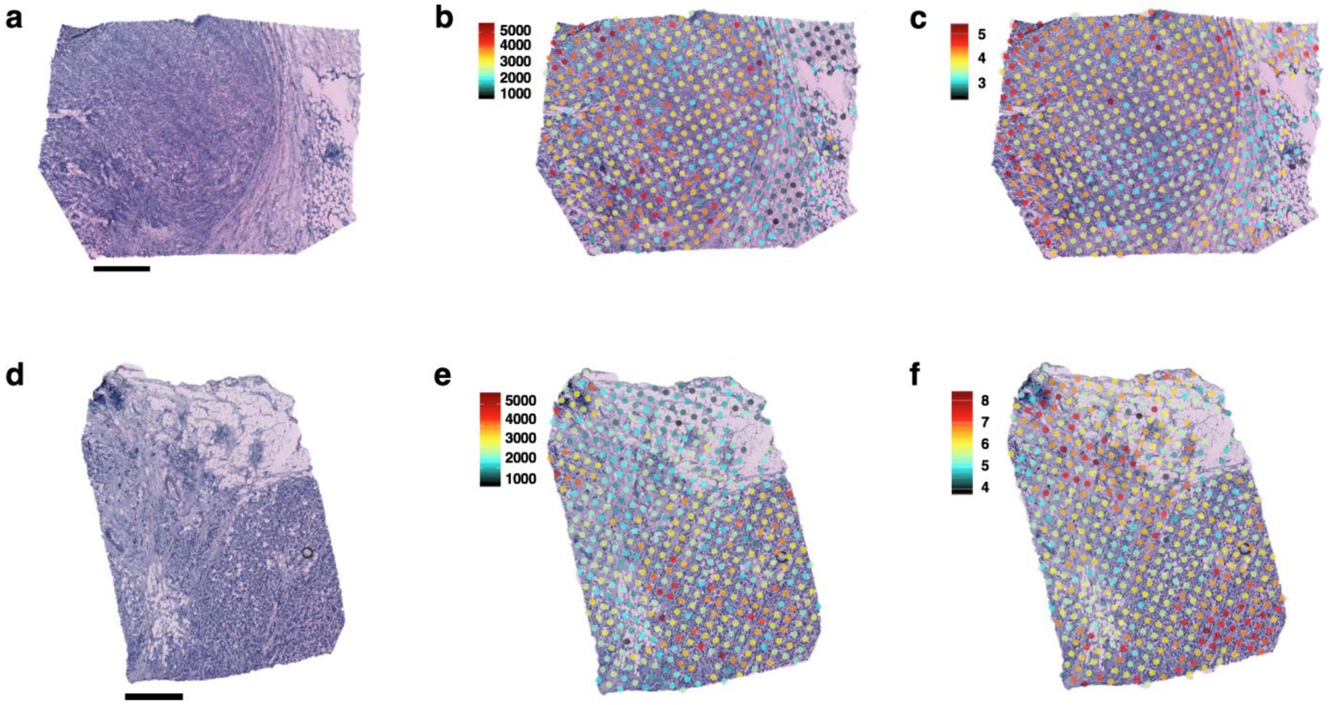

Supplementary Figure 7. ST analysis of Luminal B breast tumor. Showing areas within and under tissue. **a** HE image (Scale bar: 1mm), (**b**) ST analysis showing unique genes and (**c**) exon/intergenic mapping rate, from the same specimen shown in Figure 3a-d (n=3, technical replicates). **d** HE image (Scale bar: 1mm), (**e**) ST analysis showing unique genes and (**f**) exon/intergenic mapping rate, from the same specimen shown in Figure 3e-l (n=3, technical replicates).

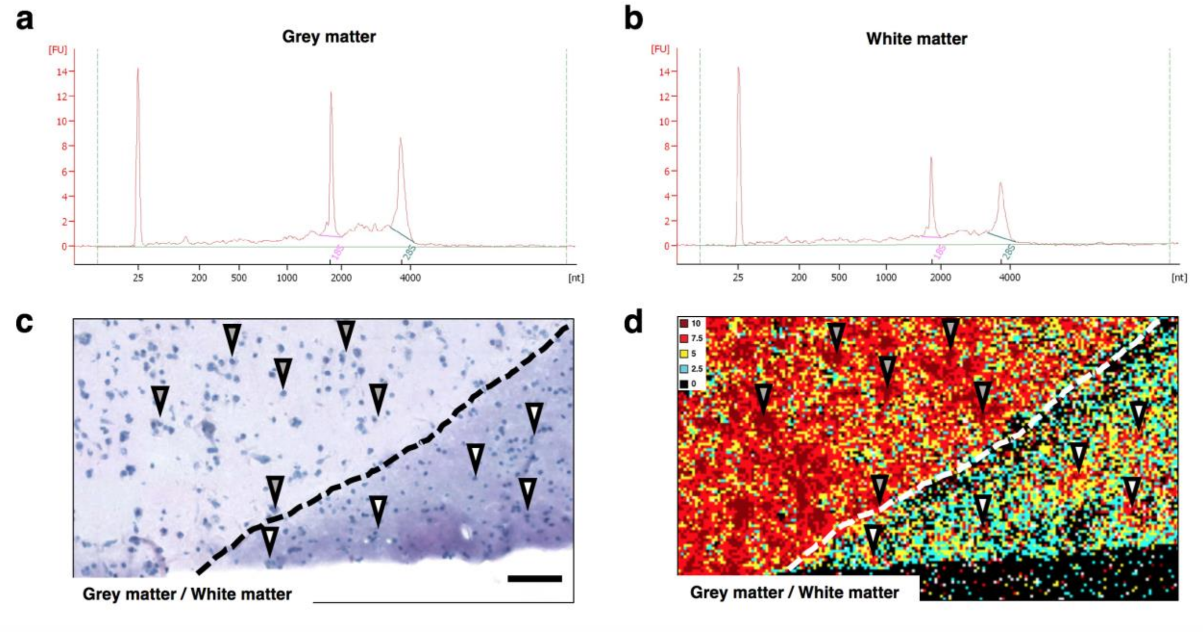

Supplementary Figure 8. RIN and sRIN heat map of post-mortem brain. Total RNA gel electropherogram for post-mortem human brain from (**a**) a grey matter region (RIN 8.0) and (**b**) a white matter region (RIN 7.8). **c** HE image (Scale bar: 100µm) and (**d**) sRIN heat map from the same specimen containing grey matter and white matter, grey arrows marks cells from grey matter regions and white arrows marks cells from white matter regions (n=3, biological replicates).

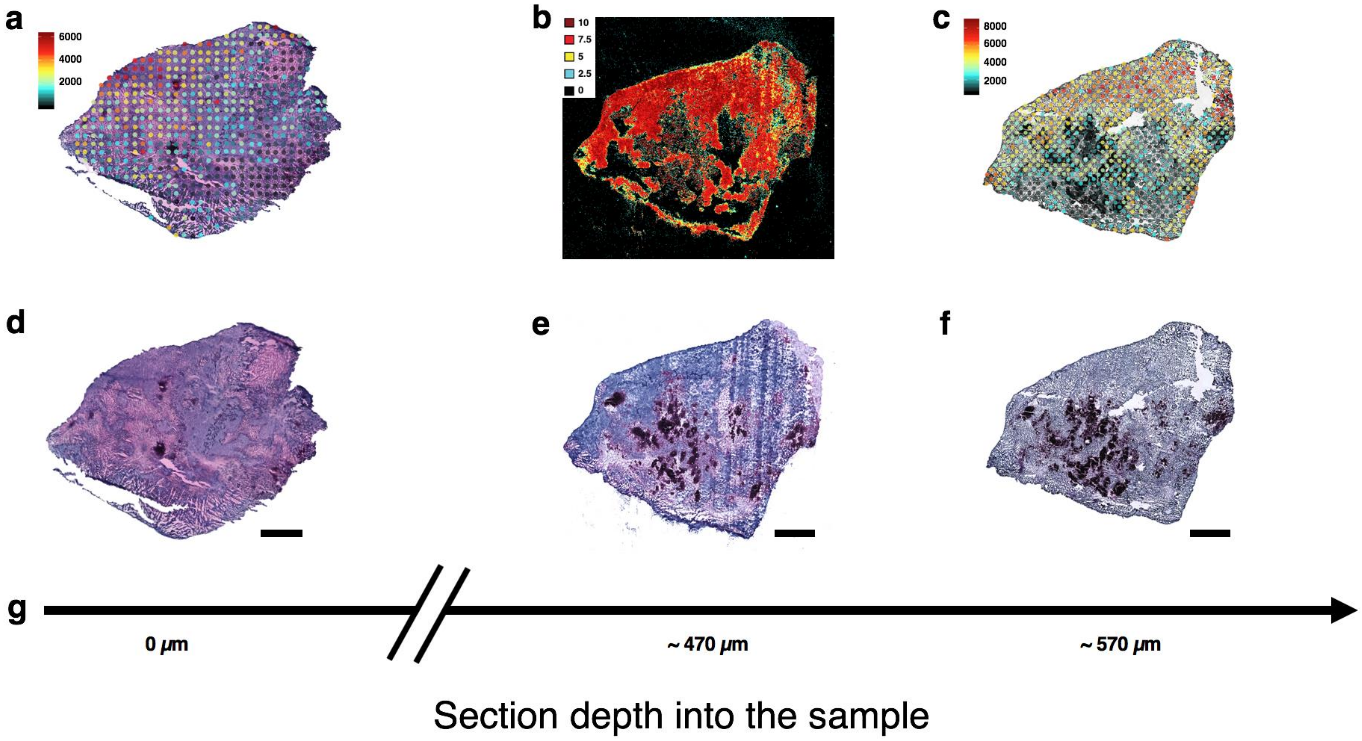

Supplementary Figure 9. Validation of the sRIN assay. Showing areas within and under tissue. ST analysis performed on sections before and after sRIN analysis of the same childhood brain tumor shown in Figure 4. **a** ST analysis showing unique genes (n=4, technical replicates). **b** sRIN heat map, also shown in figure 4 (n=4, technical replicates). **c** ST analysis showing unique genes, also shown in figure 4 (n=2, technical replicates). **d-f** corresponding HE image to (**a**, **b** and **c**), respectively (Scale bars: 1mm). **g** Axis showing different depths into the sample from the first section taken for ST analysis. By ST the number of unique genes were visualized on-top of the tissue specimens and data was generated from sections cut early on from the childhood brain tumor specimen and on serial sections cut just after the section selected for a sRIN assay. When comparing differences between the mRNA population generated using ST and RNA integrity data generated using sRIN, similar spatial patterns were observed. Larger regional RNA integrity patterns could be observed in the tissue specimen even from the earliest sections used for ST and could be followed deeper into the tissue sample.

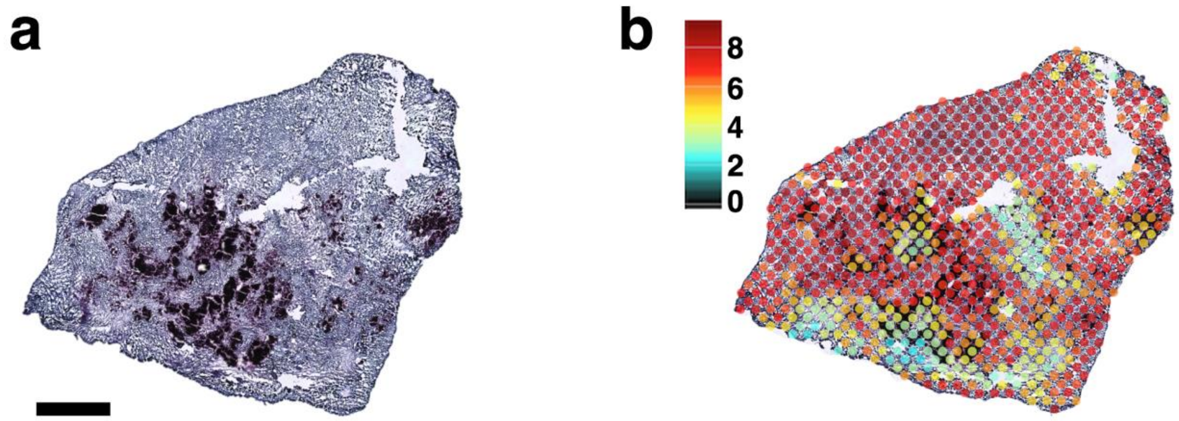

Supplementary Figure 10. The spatial exon/intergenic mapping rate.
a HE image (Scale bar: 1mm) and (**b**) ST analysis showing exon/intergenic mapping rate from the same specimen displayed in Figure 4 (n=2, technical replicates).

Supplementary Table 1. Probes and primers

Name	Sequence 5'-3'
Capture probe (sRIN slide surface)*	*UUUUUTTTACTTCCTCTAGATAGTC
Capture probe	TTTACTTCCTCTAGATAGTC
Surface probe**	**GACTATCTAGAGGAAGTAAA
Probe 1 (P1)***	***GAGGAATTCCCAGTAAGT
Probe 2 (P2)***	***GAGATTGAGCAATAACAG
Probe 3 (P3)***	***GTAGTTCCGACCATAAAC
Probe 4 (P4)***	***GGTGACTCTAGATAACCT
Reverse Primer 1	TCCTCTAGATAGTCAAGTTC
Reverse Primer 2	AATCAACGCAAGCTTATGAC
Reverse Primer 3	GTGTTGAGTCAAATTAAG
Reverse Primer 4	CGAAAGAGTCCTGTATTG
Control Probe A (P1-P3)*	*UUUUUACTTACTGGGAATTCCTCGCGTATGCG GATTGGGCTCTGTTATTGCTCAATCTCGCGTAT GCGGATTGGGCTGTTTATGGTCGGAACTAC
Control Probe B (P2-P4)*	*UUUUUCTGTTATTGCTCAATCTCGCGTATGCG GATTGGGCTGTTTATGGTCGGAACTACGCGTAT GCGGATTGGGCTAGGTTATCTAGAGTCACC

Used in the sRIN assay and during validation of the method. Regions marked in green are complementary for P1-P4, *5'-end amino-C6 modification, **5'-end Cy5 fluorophore and ***5'-end Cy3 fluorophore.

Supplementary Table 2. Specimen Bioanalyzer RIN values

Specimens	RIN
Breast cancer, Luminal B, patient 1 (Fig. 3a-d)	7.5
Breast cancer, Luminal B, patient 2 (Fig. 3e-l)	8.5
Childhood brain tumor, Anaplastic medulloblastoma Grade IV	8.4
Prostate cancer cell line LNCaP	10
Mouse olfactory bulb (Fig. 1)	10
Mouse olfactory bulb (Supplementary Fig. 1-3)	7.1
Post-mortem brain (grey matter)	8.0
Post-mortem brain (white matter)	7.8

Total RNA extracted from fresh frozen samples were evaluated in bulk on the Bioanalyzer system for RIN values.
