# Supplementary material for: The spatial RNA integrity number assay for in situ evaluation of transcriptome quality
Source: Commun Biol. 2021 Jan 8;4:57. doi: 10.1038/s42003-020-01573-1 (PMC7794352; doi:10.1038/s42003-020-01573-1)
Supplement: Supplementary file 3 — Description of Additional Supplementary Files [file 42003_2020_1573_MOESM3_ESM.pdf]

Description of additional supplementary file

File Name: Supplementary_Data_1
Description: Data used to generate graphs and charts shown in Supplementary Figures: 1b, 2, 3, 4, and 5f.
