# Supplementary material for: The spatial RNA integrity number assay for in situ evaluation of transcriptome quality
Source: Commun Biol. 2021 Jan 8;4:57. doi: 10.1038/s42003-020-01573-1 (PMC7794352; doi:10.1038/s42003-020-01573-1)
Supplement: Supplementary file 5 — Reporting Summary [file 42003_2020_1573_MOESM5_ESM.pdf]

nature research

Corresponding author(s): Joakim Lundeberg

Last updated by author(s): Oct 23, 2020

Reporting Summary

Nature Research wishes to improve the reproducibility of the work that we publish. This form provides structure for consistency and transparency in reporting. For further information on Nature Research policies, see Authors & Referees and the Editorial Policy Checklist .

Statistics

For all statistical analyses, confirm that the following items are present in the figure legend, table legend, main text, or Methods section.

n/a	Confirmed	
☐	☒	The exact sample size (*n*) for each experimental group/condition, given as a discrete number and unit of measurement
☐	☒	A statement on whether measurements were taken from distinct samples or whether the same sample was measured repeatedly
☐	☒	The statistical test(s) used AND whether they are one- or two-sided<br>*Only common tests should be described solely by name; describe more complex techniques in the Methods section.*
☐	☒	A description of all covariates tested
☐	☒	A description of any assumptions or corrections, such as tests of normality and adjustment for multiple comparisons
☐	☒	A full description of the statistical parameters including central tendency (e.g. means) or other basic estimates (e.g. regression coefficient) AND variation (e.g. standard deviation) or associated estimates of uncertainty (e.g. confidence intervals)
☐	☒	For null hypothesis testing, the test statistic (e.g. *F*, *t*, *r*) with confidence intervals, effect sizes, degrees of freedom and *P* value noted<br>*Give P values as exact values whenever suitable.*
☒	☐	For Bayesian analysis, information on the choice of priors and Markov chain Monte Carlo settings
☒	☐	For hierarchical and complex designs, identification of the appropriate level for tests and full reporting of outcomes
☐	☒	Estimates of effect sizes (e.g. Cohen's *d*, Pearson's *r*), indicating how they were calculated

Our web collection on statistics for biologists contains articles on many of the points above.

Software and code

Policy information about availability of computer code

Data collection	The present study does not involve data collection.
Data analysis	For producing sRIN heat maps: https://github.com/ludvigla/sRIN<br>For processing spatial transcriptomics sequencing data: https://github.com/SpatialTranscriptomicsResearch/st_pipeline<br>For obtaining spatial spot positions of individual spatial transcriptomics arrays: https://github.com/SpatialTranscriptomicsResearch/st_spot_detector

For manuscripts utilizing custom algorithms or software that are central to the research but not yet described in published literature, software must be made available to editors/reviewers. We strongly encourage code deposition in a community repository (e.g. GitHub). See the Nature Research guidelines for submitting code & software for further information.

Data

Policy information about availability of data

All manuscripts must include a data availability statement. This statement should provide the following information, where applicable:

- Accession codes, unique identifiers, or web links for publicly available datasets
- A list of figures that have associated raw data
- A description of any restrictions on data availability

For source data underlying the graphs and charts, see Supplementary Data 1. ST and sRIN data, such as HE images, count files, spot files and tif files with accompanied Metadata may be found here: (DOI:10.17632/kzfd6mbnxg.1)

All probe and primer sequences may be found in Supplementary Table 1 in Supplementary Information.

Raw fastq files for the breast cancer and childhood brain tumor samples are available through a Materials transfer agreement with Å.B. (ake.borg@med.lu.se) and M.N (monica.nister@ki.se), respectively, in line with GDPR regulations.

Further data supporting the findings of this study are available from the corresponding author upon reasonable request.

Field-specific reporting

Please select the one below that is the best fit for your research. If you are not sure, read the appropriate sections before making your selection.

- [x] Life sciences
- [ ] Behavioural & social sciences
- [ ] Ecological, evolutionary & environmental sciences

For a reference copy of the document with all sections, see nature.com/documents/nr-reporting-summary-flat.pdf

Life sciences study design

All studies must disclose on these points even when the disclosure is negative.

Sample size

For all spatial transcriptomics (ST) and sRIN tissue experiments: The sample size (number of tissue sections) varied from two to four, which is in the range of most previous published ST experiments. Specimens included both mouse and human, in total we applied the sRIN assay to seven distinct tissue regions.

Figure 3: The two breast cancer specimens are included in a previously published dataset (DOI: 10.17632/29ntw7sh4r.2), specifically for:
Patient 1, shown in figure 3a-d, in the previously published dataset having ID: 23277
Patient 2, shown in figure 3e-l, in the previously published dataset having ID: 23508

Supplementary Figure 1: Three technical replicates were selected for qPCR using cDNA template originating from total RNA, we expected the variability to be low and considered the selected sample size to be sufficient. For qPCR using cDNA template originating from in situ total RNA, we expected the variaility to be higher and considered the selected sample size of 18 technical replicates to be sufficient.

Supplementary Figure 2 and 3: Five biological replicates were considered enough to measure the biological variation.

Supplementary Figure 4: Six technical replicates were considered enough to show the technical variation.

Supplementary Figure 5: Five biological replicates were considered enough to measure the biological variation.

Supplementary Figure 6: Two and three biological replicates were used for the two different groups. Sample size was deemed enough to see a trend but too small for statistical inference.

Data exclusions

Supplementary Figure 1: For qPCR Cq values of in situ samples 18 technical replicates were done per probe position, Cq values scoring 0 or above 30 were excluded from the figure. The random Cq 0 values from a sample were considered technical errors and Cq values above 30 was set as a cut-off for positive signals compared to negative controls.

Replication

We applied the sRIN assay to different sample types and species, all generating sRIN heat maps showing spatial RNA quality at a single cell resolution. Experimental results demonstrate the sRIN assays ability to measure different cDNA lengths in situ. All attempts at replication were successful.

Randomization

Supplementary Figure 6: When investigating the sRIN assay's 18S rRNA specificity, when analyzing total RNA samples were divided into separate groups based on the presence or absence of a visible 18S rRNA peak in their respective gel electropherograms.

Blinding

Blinding was not relevant to this study as there were no stratified comparisons between groups.

Reporting for specific materials, systems and methods

We require information from authors about some types of materials, experimental systems and methods used in many studies. Here, indicate whether each material, system or method listed is relevant to your study. If you are not sure if a list item applies to your research, read the appropriate section before selecting a response.

Materials & experimental systems

n/a	Involved in the study	
x		Antibodies
	x	Eukaryotic cell lines
x		Palaeontology
	x	Animals and other organisms
	x	Human research participants
x		Clinical data

Methods

n/a	Involved in the study	
x		ChIP-seq
x		Flow cytometry
x		MRI-based neuroimaging

Eukaryotic cell lines

Policy information about cell lines

Cell line source(s)	LNCaP from Cell-Lines Service, Germany
Authentication	Cell line was not authenticated.
Mycoplasma contamination	Cell line was not tested for mycoplasma contamination.
Commonly misidentified lines (See ICLAC register)	Note used.

Animals and other organisms

Policy information about studies involving animals; ARRIVE guidelines recommended for reporting animal research

Laboratory animals	Mouse olfactory bulbs from C57BL/6 mice (> 2 months old) were collected, gender unknown for specific samples.
Wild animals	The study did not involve wild animals.
Field-collected samples	The study did not involve samples collected from the field.
Ethics oversight	Stockholms Djurförsöksetiska Nämnd

Note that full information on the approval of the study protocol must also be provided in the manuscript.

Human research participants

Policy information about studies involving human research participants

Population characteristics	Breast cancer, Luminal B, patient 1 (RIN 7.5)<br>Breast cancer, Luminal B, patient 2 (RIN 8.5)<br>Childhood brain tumor, Anaplastic medulloblastoma grade IV (RIN 8.4)<br>Post-mortem brain (grey matter) (RIN 8.0)<br>Post-mortem brain (white matter) (RIN 7.8)
Recruitment	Human biospecimens were selected at random.
Ethics oversight	Research was approved by the following ethical committees: etikprövningsnämnden in Stockholm, etikprövningsnämnden in Lund and the Wales Research Ethics Committee.

Note that full information on the approval of the study protocol must also be provided in the manuscript.
